# Biological hypoxia in pre-transplant human pancreatic islets induces transplant failure in diabetic mice

**DOI:** 10.1038/s41598-024-61604-3

**Published:** 2024-05-30

**Authors:** Hiroyuki Kato, Mayra Salgado, Daniel Mendez, Nelson Gonzalez, Jeffrey Rawson, Doreen Ligot, Bennie Balandran, Chris Orr, Janine C. Quijano, Keiko Omori, Meirigeng Qi, Ismail H. Al-Abdullah, Yoko Mullen, Hsun Teresa Ku, Fouad Kandeel, Hirotake Komatsu

**Affiliations:** 1 Department of Translational Research and Cellular Therapeutics, Arthur Riggs Diabetes AND Metabolism Research Institute of City of Hope, 1500 E. Duarte Rd., Duarte, CA 91010 USA; 2 Department of Surgery, University of California, San Francisco, 513 Parnassus Ave., San Francisco, CA 94143 USA

**Keywords:** Endocrinology, Type 1 diabetes, Medical research, Translational research

## Abstract

Evaluating the quality of isolated human islets before transplantation is crucial for predicting the success in treating Type 1 diabetes. The current gold standard involves time-intensive in vivo transplantation into diabetic immunodeficient mice. Given the susceptibility of isolated islets to hypoxia, we hypothesized that hypoxia present in islets before transplantation could indicate compromised islet quality, potentially leading to unfavorable outcomes. To test this hypothesis, we analyzed expression of 39 hypoxia-related genes in human islets from 85 deceased donors. We correlated gene expression profiles with transplantation outcomes in 327 diabetic mice, each receiving 1200 islet equivalents grafted into the kidney capsule. Transplantation outcome was post-transplant glycemic control based on area under the curve of blood glucose over 4 weeks. In linear regression analysis, *DDIT4* (R = 0.4971, *P* < 0.0001), *SLC2A8* (R = 0.3531, *P* = 0.0009) and *HK1* (R = 0.3444, *P* = 0.0012) had the highest correlation with transplantation outcome. A multiple regression model of 11 genes increased the correlation (R = 0.6117, *P* < 0.0001). We conclude that assessing pre-transplant hypoxia in human islets via gene expression analysis is a rapid, viable alternative to conventional in vivo assessments. This approach also underscores the importance of mitigating pre-transplant hypoxia in isolated islets to improve the success rate of islet transplantation.

## Introduction

Type 1 diabetes is an autoimmune disorder characterized by the destruction of insulin-producing pancreatic islet β-cells. Islet transplantation offers a promising potential for achieving insulin independence and ameliorating life-threatening hypoglycemia events, along with secondary organ complications in individuals with type 1 diabetes^1–3^.

A thorough assessment of isolated human islet products before transplantation is critical for predicting clinical islet transplantation success. Several factors are considered when determining if the islets are suitable for transplantation, including the condition of the donor-pancreas and recipient and in vitro parameters of the isolated islets, such as islet yield, function, and viability^4–8^. However, currently, only a few assessments can accurately predict post-transplant outcomes. For example, islet oxygen consumption, an indicator of metabolic status, positively correlates with transplantation outcomes^9–11^. Experimentally, assays for the quality of isolated islets have included flow-cytometry-based islet cellular composition and measurement of cell metabolism molecules-based adenosine triphosphate and adenosine diphosphate^12,13^; however, these assays have not been clinically realized.

Transplantation of the isolated islets into diabetic immunodeficient mice have been considered a golden standard assay to evaluate the quality of isolated islets. However, because animal studies require a long duration to assess the islet quality, they are not ideal for predicting clinical islet transplantation outcomes immediately before transplantation. Therefore, in vitro quality control assessments of isolated islets are relied upon to make the critical go/no go decision to transplant isolated islets into patients. We previously demonstrated that the basic islet quality assessments, including an imaging-based semi-automated viability assessment tool, predict the transplantation outcomes of human islets transplanted in diabetic immunodeficient mice^14^. Because islet viability is closely associated with the oxygen environment^15^, we hypothesized that biologically preexistent hypoxia in pre-transplantation islets reduces engraftment efficacy.

## Results

### Overview of the study design

**Figure 1:**
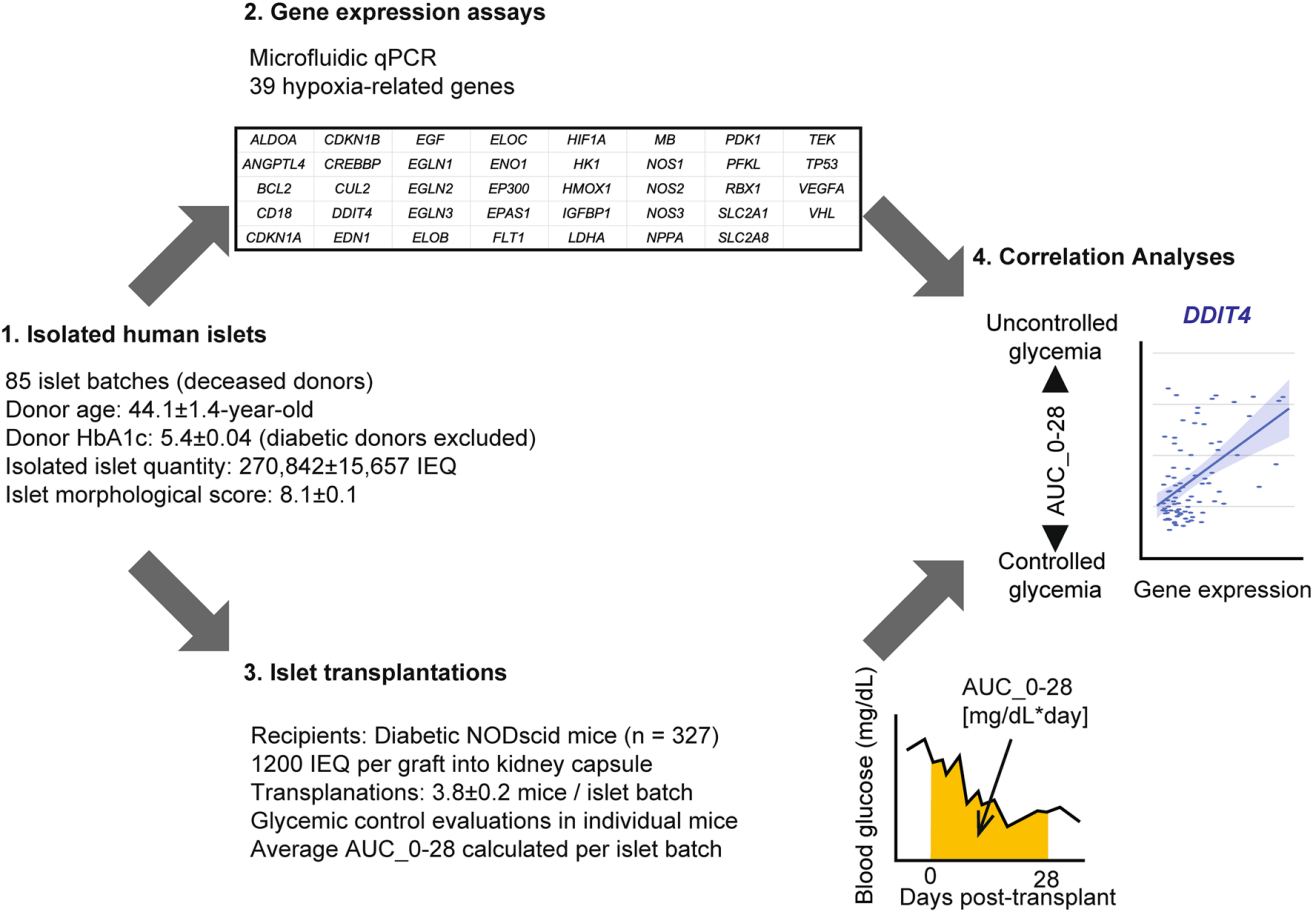
Overview of the study. The study consisted of 4 major steps: 1. Preparation of isolated islets from human deceased donors (n = 85 batches); 2. Gene expression assays of 39 hypoxia-related genes in isolated islets; 3. In vivo human islet transplantations into diabetic immunodeficiency mice (total of 327 transplantations, 3.8 mice/islet batch on average) to assess post-transplant glycemic control; and 4. Correlation analyses between gene expressions and transplantation outcomes. Data are expressed as mean ± SEM.

Figure 1 shows the overview of the design and workflow of our study, which sought to determine the correlation between the hypoxia-related gene expression in isolated islets prior to transplantation (n = 85 batches from human deceased donors) and outcomes of islet graft function after transplantation into immunodeficient diabetic mice (n = 327). Based on the literature, as well as a HIF-1signaling pathway map in the Kyoto Encyclopedia of Genes and Genomes (KEGG) database^16^, we chose the 39 hypoxia-related genes shown to be correlated with hypoxia in multiple cell types (i.e., not limited to the pancreatic islets). Table 1 provides more details.

**Table 1:** Hypoxia gene list.

Gene Symbol	Official full name	Gene ID	TaqMan Assay ID	Reference(s)
*ALDOA*	aldolase, fructose-bisphosphate A	226	Hs00605108_g1	^17^
*ANGPTL4*	angiopoietin like 4	51129	Hs00211522_m1	^18^
*BCL2*	BCL2 apoptosis regulator	596	Hs04986394_s1	^19^
*CDKN1A*	cyclin dependent kinase inhibitor 1A	1026	Hs00355782_m1	^20^
*CDKN1B*	cyclin dependent kinase inhibitor 1B	1027	Hs01597588_m1	^21^
*CREBBP*	CREB binding protein	1387	Hs00231733_m1	^22^
*CUL2*	cullin 2	8453	Hs00180203_m1	^23^
*DDIT4*	DNA damage inducible transcript 4	54541	Hs00430304_g1	^24,25^
*EDN1*	endothelin 1	1906	Hs00174961_m1	^26^
*EGF*	epidermal growth factor	1950	Hs01099999_m1	^27^
*EGLN1*	egl-9 family hypoxia inducible factor 1	54583	Hs00254392_m1	^23^
*EGLN2*	egl-9 family hypoxia inducible factor 2	112398	Hs00363196_m1	^23^
*EGLN3*	egl-9 family hypoxia inducible factor 3	112399	Hs00222966_m1	^23^
*ELOB*	elongin B	6923	Hs00793006_m1	^23,28^
*ELOC*	elongin C	6921	Hs00855349_g1	^23,28^
*ENO1*	enolase 1	2023	Hs00361415_m1	^17^
*EP300*	E1A binding protein p300	2033	Hs00230938_m1	^19^
*EPAS1*	endothelial PAS domain protein 1	2034	Hs01026149_m1	^22,29^
*FLT1*	fms related receptor tyrosine kinase 1	2321	Hs01052961_m1	^30^
*HIF1A*	hypoxia inducible factor 1 subunit alpha	3091	Hs00153153_m1	^17^
*HK1*	hexokinase 1	3098	Hs00175976_m1	^31^
*HMOX1*	heme oxygenase 1	3162	Hs00157965_m1	^32^
*IGFBP1*	insulin like growth factor binding protein 1	3484	Hs00236877_m1	^25^
*LDHA*	lactate dehydrogenase A	3939	Hs01378790_g1	^17^
*LTBR*	lymphotoxin beta receptor	4055	Hs01101194_m1	^33^
*MB*	myoglobin	4151	Hs00193520_m1	^32^
*NOS1*	nitric oxide synthase 1	4842	Hs00167223_m1	^34^
*NOS2*	nitric oxide synthase 2	4843	Hs00167248_m1	^20,34^
*NOS3*	nitric oxide synthase 3p	4846	Hs00167166_m1	^34^
*NPPA*	natriuretic peptide A	4878	Hs00383230_g1	^35^
*PDK1*	pyruvate dehydrogenase kinase 1	5163	Hs01561847_m1	^17^
*PFKL*	phosphofructokinase, liver type	5211	Hs01036347_m1	^36^
*RBX1*	ring-box 1	9978	Hs00360274_m1	^23^
*SLC2A1*	solute carrier family 2 member 1	6513	Hs00892681_m1	^17^
*SLC2A8*	solute carrier family 2 member 8	29988	Hs00205863_m1	^37^
*TEK*	TEK receptor tyrosine kinase	7010	Hs00945150_m1	^38^
*TP53*	tumor protein p53	7157	Hs00153349_m1	^20,22^
*VEGFA*	vascular endothelial growth factor A	7422	Hs00900054_m1	^23^
*VHL*	von Hippel-Lindau tumor suppressor	7428	Hs01650959_m1	^28,39^

### Hypoxia-related gene expression in pre-transplant islets correlates with post-transplant glycemic control.

**Figure 2:**
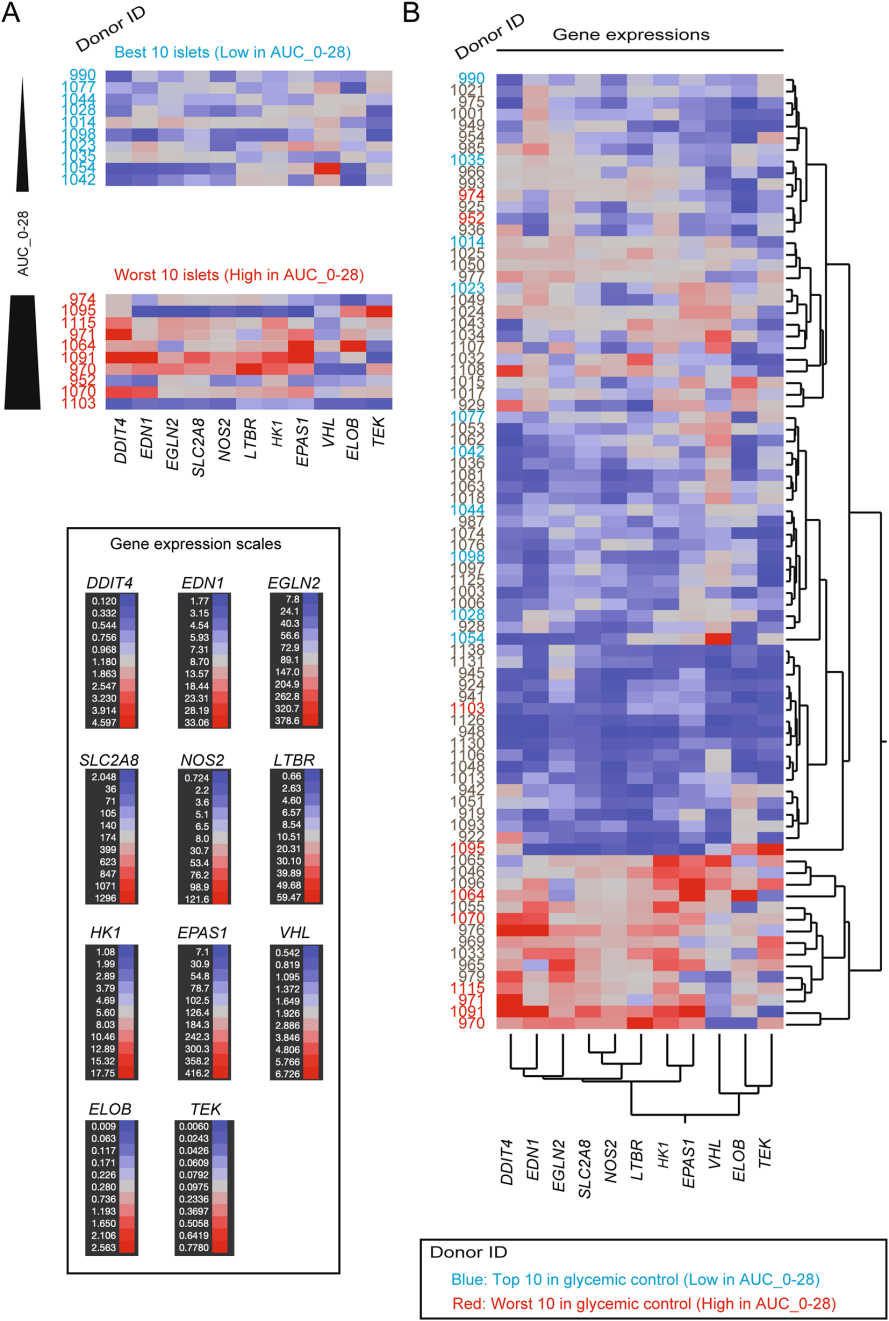
Heatmaps of the expressions of hypoxia genes. (**A**) Heatmaps are arranged according to the AUC_0–28 values. Islet batches with better post-transplant glycemic control are placed in higher rows. Donor IDs are shown in the left column; the detailed donor information is found in Table S3. Donor IDs in blue indicate the top 10 islet batches with good post-transplant glycemic control (i.e., low AUC_0–28). Donor IDs in red indicate the worst 10 islet batches with uncontrolled post-transplant glycemic control (i.e., high AUC_0–28). Bottom left: Gene expression scales for selected individual genes. (**B**) The two-way hierarchical clustering of 11 selected genes and all 85 islet batches. Donor IDs are shown in the left column.

Among the 39 genes tested, 11 were significantly correlated with post-transplant glycemic control (Table 2). Of these 11 genes, only *VHL* showed a positive correlation (R > 0) to the area under the curve (AUC) of post-transplant blood glucose (AUC_0–28), indicating that upregulation of these genes in human islets leads to unfavorable glycemic control in diabetic mice. These genes are known to be involved in the hypoxia pathway (Figure S1A). Figure 2A shows selected islet batches in order of glycemic control (AUC_0–28); islet batches with the best 10 and worst 10 glycemic controls had contrasting heatmap colors. Therefore, these hypoxia genes in human islets offer predictive insight into transplantation outcomes. Figure 2B shows the heatmap of the expression of selected genes among all 85 islet batches with two-way hierarchical clustering. Clusters showing higher expression of hypoxia-related genes except *VHL* in islet batches were correlated with uncontrolled post-transplant glycemic controls. Although genes have similarities regarding relevance to the hypoxia, the interaction analyses between the key genes demonstrated that some gene expression was independent of others in our dataset (e.g., *DDIT4* vs. *TEK*; *EDN1* vs. *VHL*, Figure S1B). Aside from the gene expression analysis, we also identified islet preparation parameters that correlate with transplantation outcomes (Table S1; see the Materials and Methods section for information on donors and isolated islets). Overall, hypoxia genes demonstrated a stronger correlation compared to islet preparation parameters, suggesting that the set of hypoxia genes could be promising for predicting outcomes.

**Table 2:** Hypoxia genes demonstrating correlations to the transplant outcome.

Gene name	Correlation (R) to post-transplant glycemic control (AUC_0–28)	*P* value
*DDIT4*	0.4971	< 0.0001
*SLC2A8*	0.3531	0.0009
*HK1*	0.3444	0.0012
*EDN1*	0.3302	0.002
*LTBR*	0.3201	0.0028
*EGLN2*	0.2988	0.0055
*NOS2*	0.2923	0.0066
*VHL*	-0.2513	0.0203
*ELOB*	0.2533	0.0208
*EPAS1*	0.2504	0.0208
*TEK*	0.2236	0.0397

### Multiple regression model of multiple hypoxia-related gene expression improves the ability to predict transplant outcome.

**Figure 3:**
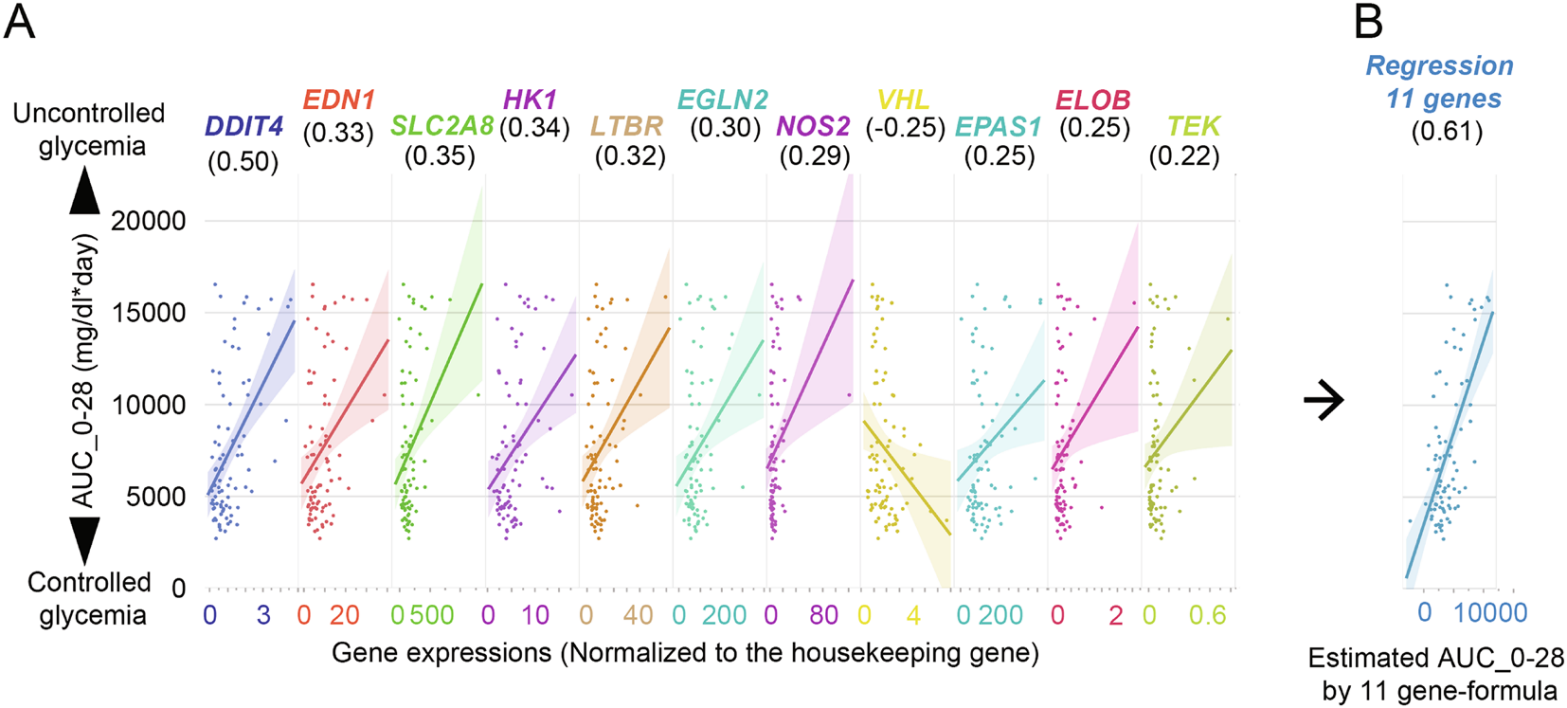
Correlation analyses of the hypoxia-related gene expression and transplantation outcome. (**A**) Correlations of individual genes to the AUC_0–28. Gene expression is shown in the x-axis, and the transplantation outcome measured using AUC_0–28 is expressed in the y-axis. All islet batches were plotted with the linear regression line. The color bands indicate the confidence of fit at a confidence interval at 95%. Correlations are shown beneath the gene names within parentheses. (**B**) Optimization of multiple regression model using 11 single gene expression data. The Estimated AUC_0–28 value calculated by the linear regression formula (Table S1) is expressed in the x-axis.

As shown in Table 2, we identified 11 key hypoxia-related genes that were significantly correlated with transplant outcome. Figure 3A shows the AUC_0–28 data plots of all islet batches against the gene expression with linear regression. To test whether these gene expression assays had potential to predict transplant outcome, we used a multiple regression model consisting of the 11 hypoxia-related genes to develop a calculation formula for the "Estimated AUC_0–28." The result was a higher correlation coefficient (R = 0.6117; *P* < 0.0001) with a narrow confidence interval, indicating better predictive capability (Fig. 3B). Further details about the linear regression formula are available in Table S2. Interestingly, a simplified multi-regression model using 3 selected genes (*DDIT4*, *HK*, and *VHL*) also had better prediction capability for transplantation outcomes than using a single gene (Figure S2, R = 0.5672, *P* < 0.0001). Further details for this linear regression formula are available in Table S3. Collectively, the regression model using expression of multiple hypoxia-related genes showed the high potential for predicting glycemic control after islet transplantation.

### The formula using expression of key hypoxia-related genes predicts islet transplantation outcomes in mice.

**Figure 4:**
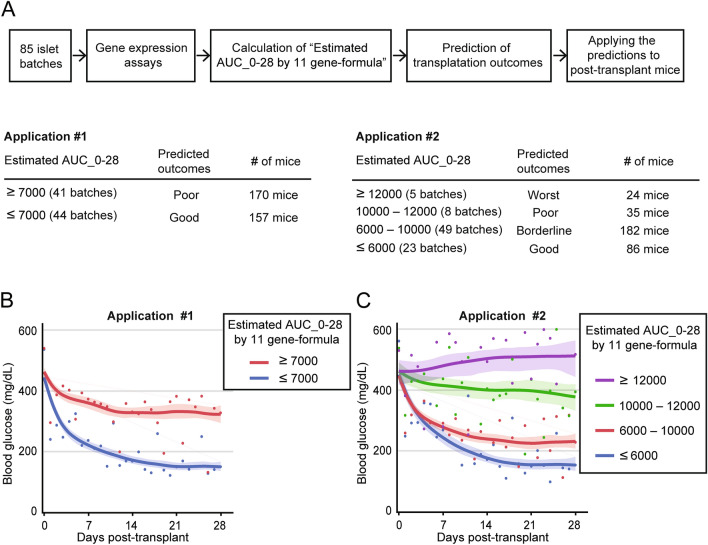
Hypoxia gene formula-based prediction models of the human islet transplantation outcomes in mice (**A**) The flowchart (top panel) outlines the formula-based outcome predictions for 85 islet batches using 11 key hypoxia-related genes. Two representative applications are shown based on "Estimated AUC_0–28" values: Application #1 predicts post-transplantation outcomes for two groups, and Application #2. predicts outcomes for four detailed groups. Bottom panels illustrate detailed information including cutoffs of AUC_0–28, predicted outcomes, and # of mice. (**B**) Two-group outcome prediction with a cutoff at 7000 mg/dl*days. (**C**) Four-group outcome prediction with multiple cutoffs. Average blood glucose data of mice are plotted in each group. Smoothers using the spline method (lambda = 2) with the confidence of fit are shown.

Using the formula based on the 11 key hypoxia-related genes, we calculated the "Estimated AUC_0–28" in post-transplant mice for 85 islet batches. The two representative applications were (1) predicting post-transplantation outcomes for two groups based on the blood glucose changes (i.e., good and poor, using the single cutoff Estimated AUC_0–28 value), and (2) predicting post-transplantation outcomes for four groups (i.e., good, borderline, poor, and worst, based on the multiple cutoff Estimated AUC_0–28 values). Figure 4A illustrates the flowchart of the assessments, including details of the settings, such as cutoffs for the Estimated AUC_0–28 values for the outcome groups and the number of islet batches and mice in each group. The two-group outcome prediction, utilizing the cutoff of Estimated AUC_0–28 at 7000 mg/dl*days, distinctly separated good and poor post-transplantation blood glucose changes in mice (Fig. 4B). More significantly, the four-group outcome prediction yielded four distinct curves of post-transplant glucose changes (Fig. 4C). Given that the formula calculates the Estimated AUC_0–28 as numerical values, we can predict the post-transplantation outcomes that are borderline cases with substantial decreased blood glucose, but not reversal of diabetes, such as AUC_0–28 values between 6000 and 10,000 (Fig. 4C, Figure S3).

## Discussion

The major limitation of using an animal model to predict clinical islet transplantation success has been the inability to acquire animal islet data expeditiously before a patient is to undergo islet transplantation. Our study demonstrates that preexistent hypoxia in pre-transplant isolated human islets predicts poor islet transplantation outcomes in diabetic mice, and that using multiple gene expression results increases the prediction potential of transplantation outcomes. These findings suggest that assessing hypoxia-related genes with conventional qPCR is a promising approach to predict animal transplantation outcomes within just in few hours. 

The hypoxia gene signature of murine islets was previously shown by exposing the isolated islets to hypoxic conditions before transplantation, and expression of these genes was correlated with the poor glycemic control in a syngeneic islet transplantation model^40^. Importantly, our study demonstrates that the hypoxia-related genes are expressed in isolated human islets even in conventional culture environments, and preexistent intra-islet hypoxia is associated with unfavorable transplantation outcomes. Another pioneering work, Kurian et. al identified 262 genes as potential biomarkers for successful islet transplantations in immunodeficient mice; these genes are biologically associated with inflammation and repair mechanisms^41^. Their probe set classifiers did not include the hypoxia-related genes identified in our study. Our study differs from that of Kurian et. al.’s in two critical ways: the number of genes examined and the method for evaluating transplantation outcome. Kurian et. al. used whole genome analysis and a non-hypothesis-driven approach, whereas our study was hypothesis-driven and focused on the hypoxia-related genes using microfluidic qPCRs. To evaluate outcome, Kurian et. al. used reversal of diabetes, a yes and no-type qualitative assessment that has been used frequently. We used quantitative values (AUC_0–28) for the post-transplant glycemic controls, which allowed us to perform regression analyses to evaluate the correlations between post-transplant glycemic controls and gene expressions in 85 islet batches. Kurian et. al. performed validations using 16 islet batches for the selected genes after the first discovery step. Next-generation sequencing analyses, such as RNA sequencing, are the ideal approach because of their high discovery power suitable for the hypothesis-free approach to detect novel genes for new predictive biomarkers. On the other hand, the advantage of our method of microfluidic qPCRs is that the accuracy and sensitivity of the results is better than those from next-generation sequencing analyses.

We identified 11 key genes that were significantly correlated with successful transplantation outcomes. The majority of them were hypoxia-inducible-factor target genes, including *LTBR, EDN1, NOS2, HK1, and TEK* that correlated positively with inadequate post-transplant glycemic control, indicating that upregulation of these genes in pre-transplant islets leads to post-transplantation failures*.* Previous studies demonstrated that *LTBR* recruits leucocytes, which may have a causal role in islet destruction^42^; increased *EDN1* correlates to glucose intolerance and insulin resistance in mice with high-fat diet as well as intermittent hypoxia^43,44^; *NOS2,* a known mediator of beta cell dysfunction, is also a target of NF-κB proinflammatory signaling pathway^45^; *HK1* impairs glucose metabolism to induce high basal insulin secretion from the beta cells^46^; *TEK,* an angiopoietin receptor, and its ligands are involved in the diabetes progression^47^. Interestingly, among the genes tested, only *VHL* had a significant negative correlation with the AUC_0–28 (R = -0.2549; i.e., upregulation of *VHL* is correlated with good post-transplant glycemic control). *VHL* is involved in the ubiquitination and prolyl hydroxylation-initiated degradation of *HIF1A*. In fact, it was reported that the loss of *VHL* induced impaired β-cell function and mass via *HIF1A* upregulation^48,49^.

*DDIT4 (REDD1)* had the strongest correlation with islet transplantation outcome as a single gene in our study, followed by *SLC2A8*. *DDIT4* represses the mammalian or mechanistic Target of Rapamycin (mTOR)^50^, which, in theory, could reduce islet graft survival. On the other hand, *DDIT4* is multifaceted and increases insulin production^51^. *SLC2A8* encodes glucose transporters. Although the correlation between the *SLC2A8* and islets has not been well documented, our results suggested it has a detrimental effect on transplantation outcome. A previous study found that hypoxia downregulated *SLC2A8* in the epithelial cells in vitro, in a manner independent from the *HIF1A* pathway^37^.

Alterations in hypoxia-related genes are known to affect various biological functions, including inflammation, apoptosis, angiogenesis, and promoting anaerobic metabolism. Inducing inflammation may damage the islet graft, whereas the upregulation of angiogenesis is requisite for graft revascularization. However, in our study, rather than having a beneficial effect in diabetic mice, hypoxia-related gene alterations induced negative transplantation outcomes. Our result suggests that the improved oxygen environment of the pre-transplanted islets prevents hypoxia gene alterations and could enhance transplantation success. Establishing optimal oxygenated conditions for pre-transplanted islets by modifying culture settings is a promising translational approach^52–55^.

In addition to its primary application to predict the human islet transplantation outcome in mouse recipients, another prospective use of the hypoxia-related genes lies in direct prediction of clinical outcomes of islet transplantations. This includes the current use of human islets from deceased donors, the potential use of xenogeneic islets, and stem-cell-derived islets^56^. The key genes identified in our study could potentially be used as predictive markers. Moreover, a multiple regression model may be more predictive than single-gene models for decision-making in clinical islet transplantations. Nevertheless, it is important to validate and reassess the gene set in a large cohort study, incorporating additional hypoxia-related genes that may exhibit improved correlation with clinical islet transplantation outcomes from various sources. It is crucial to note that hypoxia resistance may vary depending on the sources, affecting the predictive ability of this hypoxia genes model on transplantation outcomes.

## Materials and methods

### Human donor pancreata for islet isolation

Human islets were isolated from human pancreata obtained from deceased donors through OneLegacy, a local organ procurement organization in the greater Los Angeles area^57^. Islets were isolated the Southern California Islet Cell Resource Center (SC-ICRC) at City of Hope, following standard operating procedures. All pancreata processed in this study were approved for research by the Institutional Review Board of City of Hope (IRB # 01046), and informed consent was obtained from family or relatives of the donors.

SC-ICRC at City of Hope procured 220 cases of human islet isolations between October 2014 and September 2018, and the RNA samples of 90 cases were obtained for this study. Islet batches from donors with glycated hemoglobin (HbA1c) > 6.5% were excluded. Islet batches transplanted into only a single mouse were also excluded. In total, 85 islet batches met the inclusion criteria. There were 22 females and 63 males, the average donor age was 44.1 ± 1.4 years, and the average donor HbA1c was 5.4 ± 0.04%. Standardized characteristics, consistent with the recommendations of *Diabetes* and *Diabetologia*^58,59^, are summarized for donors and islets used in this study in Supplementary information, Table S4.

### Isolation of human islets and post-isolation assessments

Islets were isolated as described previously^60–62^. The following post-isolation assessments were then performed: islet count (post-isolation islet equivalent number [IEQ] and post-isolation islet particulate number [IPN]), multiparametric morphological assessment^62^, islet purity (by dithizone [DTZ] stain), and viability (conventional and semi-automated methods; Table S4)^6,14^. Note that the semi-automated viability data was available only for 45 islet batches in this dataset.

### Gene expression assays of isolated human islets

RNA was isolated from 500 IEQ of islets to synthesize cDNAs according to the manufacturer’s instructions (cDNA Synthesis Kit for RT-qPCR; Thermo Fisher Scientific, Waltham, MA). Microfluidic quantitative real-time PCR (qRT-PCR) was performed using the BioMark 48.48 Dynamic Array system (Standard Biotools, South San Francisco, CA). Genes previously demonstrating correlations to hypoxia, not limited to the pancreatic islet research, were chosen; TaqMan probes (Life Technologies, Carlsbad, CA) used in the qRT-PCR are listed in Table 1. The expressions of the genes of interest were normalized to the internal control gene (*GAPDH*). Relative quantities of each transcript were expressed as a fold increase to the internal control gene.

### Human islet transplantation into diabetic immunodeficient mice

8–12-week-old non-obese diabetic, severe combined immunodeficiency (NODscid) male mice (Charles River Laboratories, Wilmington, MA) were used as recipients for islet transplantations as described^14,63^. Mice were rendered diabetic by intraperitoneal injections of 50 mg/kg of streptozotocin (Sigma-Aldrich, St. Louis, MO) for three consecutive days. Mice with a blood glucose level > 400 mg/dL were used for the transplantation of human islets. Blood glucose levels were monitored twice a week. Diabetes reversal was defined as blood glucose level < 200 mg/dL on at least three consecutive measurements. A total of 327 mice were transplanted with isolated human islets from 85 separate isolations (human donors). For each islet isolation, the average of 3.8 ± 0.2 mice/islet batch (range: 2–8 mice) were transplanted with 1200 IEQ under the renal capsule. All transplantation procedures were performed under general anesthesia using 2–3% isoflurane (Dechra, Northwich, United Kingdom) followed by the subcutaneous administration of an analgesic (Buprenorphine extended-release injection at 1 mg/kg, Wedgewood Pharmacy, AZ) immediately after the surgical procedures. Mice were closely monitored for > 1 month, and blood glucose levels were measured twice a week using a glucose meter (LifeScan, Inc., Malvern, PA). To analyze post-transplant blood glucose control quantitatively, AUC for days 0–28 (AUC_0–28) was calculated using the blood glucose measurements from days 0–28, which reflects glucose change for 4 weeks in an individual mouse^14^. This assessment allows us to evaluate more detailed glycemic control, rather than the conventional qualitative diabetes reversal assessment using the specific blood glucose cut-off value (Figure S3). Finally, the average of AUC_0–28 was calculated within the experimental set of each islet donor, which represented the in vivo post-transplant glycemic control ability of the islet batch. AUC_0–28 data of each islet batch is found in Table S4. At the end of the experiments, mice were euthanized using carbon dioxide inhalation. Institutional Animal Care and Use Committee at City of Hope/Beckman Research Institute approved the use of animals and animal procedures in this study (IACUC #01020), and all experiments were performed in accordance with relevant guidelines and regulations.

### Statistics

Statistical analysis was performed using JMP 16 (SAS Institute, Cary, NC). Donor characteristics and isolated islet data are reported as mean ± standard error of the mean. Multivariate analysis was performed to identify the statistical correlations (R) between several variables (Pearson’s correlation). Expressions of the key genes in all islet batches were presented in the heat maps. The similarities of islet batches in the expressions of the key genes were demonstrated in Ward’s hierarchical clustering with dendrograms. Linear regression was performed to determine correlations between gene expression and the transplantation outcome measured in AUC_0–28. All data points were plotted with the linear regression line and the color bands for the confidence of fit (a confidence interval at 95%). Comparisons between the two variables were analyzed using Student’s t-test, with values of *P* < 0.05 considered significant.

### Ethics approval

Human pancreata of deceased donors processed in this study were approved for research by the Institutional Research Board of City of Hope (IRB # 01046), and informed consent was obtained from family or relatives of the donors.

### ARRIVE guidelines

This study was reported in accordance with the ARRIVE guidelines (https://arriveguidelines.org).

### Supplementary Information

Supplementary Information.

## Data Availability

The data that support the findings of this study are available from the corresponding author upon reasonable request.

## Abbreviations

AUC: Area under the curve
DTZ: Dithizone
HbA1c: Glycated hemoglobin
IEQ: Islet equivalent
IPN: Islet particulate number
mTOR: Mechanistic target of rapamycin
NODscid: Non-obese diabetic, severe combined immunodeficiency
qRT-PCR: Quantitative real-time PCR
SC-ICRC: Southern California Islet Cell Resource Center
