# Supplementary material for: Biological hypoxia in pre-transplant human pancreatic islets induces transplant failure in diabetic mice
Source: Sci Rep. 2024 May 30;14:12402. doi: 10.1038/s41598-024-61604-3 (PMC11137081; doi:10.1038/s41598-024-61604-3)
Supplement: Supplementary file 1 — Supplementary Information. [file 41598_2024_61604_MOESM1_ESM.pdf]

Supplementary information

Title: Biological hypoxia in pre-transplant human pancreatic islets induces transplant failure in diabetic mice

Author listing and Correspondence information:

Hiroyuki Kato[1,2], Mayra Salgado[1], Daniel Mendez[1], Nelson Gonzalez[1], Jeffrey Rawson[1], Doreen Ligot[1], Bennie Balandran[1], Chris Orr[1], Janine C. Quijano[1], Keiko Omori[1], Meirigeng Qi[1], Ismail H. Al-Abdullah[1], Yoko Mullen[1], Hsun Teresa Ku[1], Fouad Kandeel[1], Hirotake Komatsu[1,2]

1. Department of Translational Research & Cellular Therapeutics, Arthur Riggs Diabetes & Metabolism Research Institute of City of Hope, 1500 E. Duarte Rd., Duarte, CA 91010, USA.

2. Department of Surgery, University of California, San Francisco, 513 Parnassus Ave. San Francisco, CA 94143, USA

* Corresponding author: Hirotake Komatsu, hirotake.komatsu@ucsf.edu

Table S1. Factors of Isolated Islets Affecting the Transplantation Outcomes

	Correlation (R) to post-transplant glycemic control (AUC_0-28)	P-value
Stimulation Index	0.2838	0.0098
Post isolation IPN	-0.298	0.0056
Islet Score	-0.2745	0.0110
Purity (%)	-0.2409	0.0264
Post isolation IEQ	-0.2366	0.0293

Table S2. Coefficient and Y-intercept of the linear regression formula

Gene	**Coefficient**
VHL	-1287
DDIT4	1393
HK1	404.9
EDN1	-125.4
TEK	3978
EGLN2	-12.77
ELOB	1230
LTBR	64.14
NOS2	27.89
EPAS1	-1.665
SLC2A8	-0.3411
Y-intercept	6985

Table S3. Coefficient and Y-intercept of the simplified linear regression formula

Gene	**Coefficient**
DDIT4	1222.64
HK1	308.44
VHL	-1060.39
Y-intercept	6384.41

Table S4. Information of donors and isolated islets

Donor_ID	Donor age (years)	Donor sex (M/F)	Donor BMI (kg/m2)	Donor HbA1c	Islet isolaton center	Cause of death of the donor	Warm ischemia time (h)	Cold ischemia time (h)	Purity (%)	Viability (%)	Total culture time (h)	Stimulation Index in GSIS	Isolation Date (MM/DD/YY)	Post isolation IEQ	Post isolation IPN	Post isolation IEQ/IPN	IsletScore	IsletGrade	Viability (semi-automated)	Post-transplant glycemic control in mice (average AUC 0-28, mg/dl*day)
919	38	M	27.6	4.7	SC-ICRC	Cerebrovascular / Stroke	N/A	4	75	97.0	71.8	1.0	10/2/14	242,867	390,000	0.6	10	A = 9 to 10 points	N/A	4,517
921	52	F	31.5	5.0	SC-ICRC	Cerebrovascular / Stroke	N/A	5	80	98.0	66.2	2.5	10/24/14	365,217	586,000	0.6	9	A = 9 to 10 points	N/A	7,700
922	53	M	34.9	6.0	SC-ICRC	Cerebrovascular / Stroke	N/A	6	80	97.0	29.5	1.2	10/29/14	299,575	217,500	1.4	8	B = 7 to 8 points	N/A	5,249
924	48	M	30.4	5.8	SC-ICRC	Anoxia / cardiovascular	N/A	5	85	98.0	N/A	0.6	11/9/14	363,783	445,500	0.8	8	B = 7 to 8 points	N/A	4,351
925	55	M	29.5	5.3	SC-ICRC	Head Trauma	N/A	7	78	97.0	N/A	0.3	11/12/14	321,599	220,000	1.5	7	B = 7 to 8 points	N/A	3,791
928	26	M	28.6	4.8	SC-ICRC	Head trauma / GSW	N/A	5	70	89.0	N/A	0.2	12/7/14	216,766	149,000	1.5	9	A = 9 to 10 points	N/A	10,457
929	43	M	29.5	5.8	SC-ICRC	Cerebrovascular / Stroke	N/A	5	80	98.4	N/A	N/A	1/3/15	369,399	185,500	2.0	10	A = 9 to 10 points	N/A	9,997
936	39	M	26.4	5.7	SC-ICRC	Cerebrovascular / Stroke	N/A	6	83	96.0	42.4	6.6	3/16/15	259,317	85,000	3.1	7	B = 7 to 8 points	N/A	11,150
941	52	M	32.4	5.0	SC-ICRC	Cerebrovascular / Stroke	N/A	6	70	97.0	78.0	5.5	4/10/15	326,650	319,000	1.0	9	A = 9 to 10 points	N/A	11,134
942	50	F	21.5	4.9	SC-ICRC	Cerebrovascular / Stroke	N/A	12	85	96.5	N/A	N/A	5/1/15	143,033	204,000	0.7	9	A = 9 to 10 points	99.38	4,370
945	53	M	21.8	5.5	SC-ICRC	Head Trauma / Blunt injury	N/A	6	85	97.4	50.3	1.5	5/24/15	516,466	490,500	1.1	9	A = 9 to 10 points	99.85	6,421
948	23	M	33.7	4.9	SC-ICRC	Anoxia	N/A	13	85	95.2	53.0	3.5	6/8/15	398,567	181,000	2.2	9	A = 9 to 10 points	N/A	7,109
949	39	M	29.2	5.6	SC-ICRC	Head Trauma / Blunt injury	N/A	5	80	98.0	42.5	1.2	6/15/15	251,316	171,000	1.5	8	B = 7 to 8 points	92.36	7,025
952	36	M	29.0	4.1	SC-ICRC	Head Trauma / MVA / Blunt injury	N/A	5	80	97.0	45.5	0.1	7/19/15	290,100	114,500	2.5	8	B = 7 to 8 points	N/A	15,860
954	33	F	23.5	4.9	SC-ICRC	Head Trauma / MVA / Blunt injury	N/A	4	78	90.0	136.0	2.4	7/29/15	237,867	353,500	0.7	8	B = 7 to 8 points	93.72	5,284
965	24	M	50.5	5.6	SC-ICRC	Cerebrovascular / Stroke	N/A	6	80	94.0	35.3	3.0	11/1/15	364,300	314,000	1.2	7	B = 7 to 8 points	N/A	5,479
966	20	M	30.8	5.6	SC-ICRC	Head Trauma / Blunt injury / Homicide	N/A	10	80	92.0	94.0	3.8	11/5/15	199,133	289,000	0.7	8	B = 7 to 8 points	N/A	13,127
969	40	M	38.3	5.5	SC-ICRC	Cerebrovascular / Stroke	N/A	11	80	95.0	31.2	2.4	11/18/16	296,283	252,500	1.2	8	B = 7 to 8 points	N/A	11,305
970	25	M	25.3	5.3	SC-ICRC	Anoxia	N/A	5	78	94.0	91.7	6.9	12/10/16	95,133	55,500	1.7	7	B = 7 to 8 points	97.37	15,829
971	47	M	31.7	4.7	SC-ICRC	Cerebrovascular / Stroke	N/A	8	83	93.8	121.6	4.2	12/17/16	321,400	290,500	1.1	9	A = 9 to 10 points	N/A	15,301
974	52	M	33.1	6.2	SC-ICRC	Head Trauma / Blunt injury	N/A	5	70	96.0	18.0	N/A	1/13/16	116,983	107,500	1.1	7	B = 7 to 8 points	N/A	14,113
975	63	M	32.0	5.8	SC-ICRC	Cerebrovascular / Stroke	N/A	5	83	95.0	102.4	4.8	1/15/16	217,017	322,000	0.7	9	A = 9 to 10 points	N/A	4,558
976	41	F	19.1	4.7	SC-ICRC	Anoxia	10	7	75	95.3	45.4	6.9	1/17/16	80,683	68,000	1.2	9	A = 9 to 10 points	N/A	9,100
977	60	M	23.7	5.7	SC-ICRC	Cerebrovascular / Stroke	N/A	7	80	95.3	64.3	2.0	1/22/16	159,950	130,000	1.2	9	A = 9 to 10 points	N/A	6,420
979	47	M	34.1	5.4	SC-ICRC	Anoxia	N/A	7	70	94.3	71.1	1.5	1/29/16	33,833	26,500	1.3	5	C = 4 to 6 points	N/A	13,806
985	52	M	25.4	5.5	SC-ICRC	Cerebrovascular / Stroke	N/A	4	70	98.2	39.8	1.6	4/24/16	62,783	65,500	1.0	8	B = 7 to 8 points	N/A	7,209
987	37	M	30.1	5.3	SC-ICRC	Head Trauma	N/A	7	70	97.8	29.1	1.3	4/26/16	247,817	166,000	1.5	9	A = 9 to 10 points	97.10	4,696
990	57	M	23.0	5.7	SC-ICRC	Head Trauma	N/A	4	85	94.3	147.3	1.6	5/25/16	366,617	417,500	0.9	9	A = 9 to 10 points	96.12	2,691
993	45	F	23.1	5.3	SC-ICRC	Cerebrovascular / Stroke	22	6	85	95.0	97.0	2.3	6/9/16	115,783	182,500	0.6	8	B = 7 to 8 points	80.39	10,005
1001	46	F	42.9	5.6	SC-ICRC	CVA / ICH	22	7	75	98.0	68.0	1.9	8/5/16	163,750	123,000	1.3	7	B = 7 to 8 points	63.53	13,404
1003	63	F	27.6	4.6	SC-ICRC	Cerebrovascular / Stroke	N/A	5	85	97.0	8.5	1.2	8/9/16	237,433	319,000	0.7	9	A = 9 to 10 points	91.56	7,928
1006	47	M	30.9	6.3	SC-ICRC	Anoxia / drowning	N/A	7	83	96.0	66.7	2.6	8/21/16	552,066	331,000	1.7	9	A = 9 to 10 points	95.49	11,700
1013	35	M	27.4	5.4	SC-ICRC	Cerebrovascular / Stroke	N/A	8	80	95.8	18.5	1.3	9/26/16	185,863	260,700	0.7	8	B = 7 to 8 points	97.66	4,583
1014	15	M	24.5	5.1	SC-ICRC	Head Trauma / GSW	N/A	5	80	94.0	70.7	2.5	10/1/16	269,867	231,000	1.2	8	B = 7 to 8 points	N/A	3,440
1015	17	M	39.4	5.2	SC-ICRC	Head Trauma	N/A	4	78	98.0	15.2	1.6	10/4/16	215,317	320,500	0.7	8	B = 7 to 8 points	96.46	4,370
1017	46	F	30.1	5.6	SC-ICRC	Cerebrovascular / Stroke	N/A	9	75	99.0	19.7	2.7	10/25/16	98,450	169,500	0.6	7	B = 7 to 8 points	86.98	6,849
1018	65	F	32.3	5.7	SC-ICRC	Anoxia	N/A	6	85	99.0	44.8	1.3	10/31/16	196,417	230,500	0.9	8	B = 7 to 8 points	N/A	6,486
1021	46	M	21.4	5.8	SC-ICRC	Head Trauma	N/A	5	75	97.5	44.8	1.6	11/12/16	226,083	201,500	1.1	8	B = 7 to 8 points	84.25	5,837
1023	50	F	21.6	5.5	SC-ICRC	Cerebrovascular / Stroke	N/A	7	80	99.0	22.4	1.6	11/28/16	219,750	416,000	0.5	8	B = 7 to 8 points	98.26	3,485
1024	48	F	31.0	5.5	SC-ICRC	Cerebrovascular / Stroke	N/A	5	80	98.0	28.1	1.5	11/29/16	190,917	347,500	0.5	9	A = 9 to 10 points	97.06	8,264
1025	33	M	29.8	5.2	SC-ICRC	Cerebrovascular / stroke	N/A	9	70	98.0	99.8	2.4	12/1/16	118,567	167,500	0.7	7	B = 7 to 8 points	96.33	7,696
1028	59	M	30.8	5.4	SC-ICRC	Cerebrovascular / Stroke	N/A	5	80	96.0	34.8	1.2	12/18/16	636,066	562,500	1.1	9	A = 9 to 10 points	95.50	3,324
1032	47	M	34.1	5.6	SC-ICRC	Anoxia	23	5	70	98.0	24.8	2.7	1/24/17	296,583	317,500	0.9	8	B = 7 to 8 points	N/A	4,477
1033	57	F	26.0	5.4	SC-ICRC	Anoxia / cardiovascular	N/A	8	80	92.2	93.6	1.9	1/26/17	279,733	324,500	0.9	9	A = 9 to 10 points	N/A	13,021
1034	65	F	35.1	5.6	SC-ICRC	Cerebrovascular / Stroke	N/A	8	80	98.0	24.0	1.1	2/15/17	432,133	618,500	0.7	9	A = 9 to 10 points	N/A	7,783
1035	24	F	33.9	5.2	SC-ICRC	Cerebrovascular / Stroke	N/A	5	80	95.7	97.4	1.5	2/22/17	330,633	191,500	1.7	9	A = 9 to 10 points	N/A	3,605
1036	60	M	31.2	5.7	SC-ICRC	Head Trauma	N/A	14	80	96.0	71.8	1.4	2/26/17	606,457	406,250	1.5	8	B = 7 to 8 points	N/A	4,964
1041	48	F	34.4	5.7	SC-ICRC	Cerebrovascular / Stroke	N/A	3	70	96.1	99.4	2.7	3/15/17	247,067	280,000	0.9	8	B = 7 to 8 points	95.12	10,498
1042	44	M	31.1	5.2	SC-ICRC	Head Trauma	N/A	6	80	94.0	37.6	2.4	3/19/17	400,466	276,500	1.4	9	A = 9 to 10 points	N/A	3,689
1043	51	F	23.0	5.8	SC-ICRC	Cerebrovascular / Stroke	N/A	6	75	97.0	66.1	1.5	3/24/17	152,300	169,500	0.9	7	B = 7 to 8 points	N/A	5,580
1044	32	M	29.1	5.6	SC-ICRC	Head Trauma	N/A	5	80	93.0	88.5	2.8	4/6/17	334,517	451,500	0.7	9	A = 9 to 10 points	N/A	3,152
1046	38	M	26.3	5.2	SC-ICRC	Head Trauma	N/A	14	75	96.0	45.2	1.7	4/9/17	223,550	252,500	0.9	7	C = 4 to 6 points	N/A	8,661
1048	53	M	29.0	5.5	SC-ICRC	ICH	35	6	78	94.0	53.6	1.3	4/22/17	599,767	798,500	0.8	8	B = 7 to 8 points	97.88	4,778
1049	43	M	28.2	5.6	SC-ICRC	Head Trauma	N/A	6	85	97.0	19.1	1.7	5/1/17	218,516	123,000	1.8	9	A = 9 to 10 points	96.53	3,935
1050	35	F	28.7	4.9	SC-ICRC	Cerebrovascular / Stroke	N/A	8	75	99.0	64.9	1.8	5/5/17	109,033	145,000	0.8	7	B = 7 to 8 points	95.40	7,998
1051	16	M	21.8	5.3	SC-ICRC	Head Trauma / GSW	N/A	8	80	98.0	52.6	1.4	5/12/17	165,567	127,500	1.3	9	A = 9 to 10 points	N/A	5,585

1053	64	M	32.2	5.7	SC-ICRC	Cerebrovascular / Stroke	N/A	5	80	97.0	27.1	2.1	5/20/17	242,600	302,000	0.8	9	A = 9 to 10 points	99.50	4,304
1054	35	M	22.8	5.6	SC-ICRC	Anoxia, drowning	N/A	5	75	96.0	44.0	1.2	5/28/17	130,133	82,000	1.6	8	B = 7 to 8 points	99.56	3,677
1055	56	M	47.7	5.2	SC-ICRC	Anoxia	20	5	73	93.0	103.8	1.6	6/7/17	117,250	115,000	1.0	7	B = 7 to 8 points	95.09	5,417
1062	46	M	33.2	5.5	SC-ICRC	Head Trauma	N/A	4	85	97.0	64.9	1.9	7/21/17	234,650	432,000	0.5	9	A = 9 to 10 points	99.18	4,600
1063	38	M	25.2	5.4	SC-ICRC	Cerebrovascular / Stroke	N/A	7	83	98.0	N/A	2.7	7/24/17	195,550	136,500	1.4	9	A = 9 to 10 points	99.13	8,759
1064	20	M	63.1	6.1	SC-ICRC	Head Trauma / MVA	N/A	7	75	97.0	N/A	2.2	7/25/17	58,583	31,000	1.9	7	B = 7 to 8 points	97.27	15,501
1065	50	M	39.1	5.7	SC-ICRC	Anoxia	N/A	7	85	98.0	102.4	1.2	7/26/17	659,249	418,500	1.6	9	A = 9 to 10 points	99.37	4,163
1070	57	M	28.6	5.0	SC-ICRC	Cerebrovascular / Stroke	N/A	6	75	97.0	30.8	0.7	8/26/17	65,517	89,000	0.7	7	C = 4 to 6 points	97.49	15,700
1074	61	M	27.0	5.6	SC-ICRC	Head Trauma / GSW	N/A	9	80	96.0	92.5	1.6	9/15/17	477,200	463,500	1.0	9	A = 9 to 10 points	98.69	5,207
1076	35	M	24.4	5.4	SC-ICRC	Cerebrovascular / Stroke	N/A	8	80	92.5	51.7	1.4	9/22/17	267,400	183,500	1.5	9	A = 9 to 10 points	96.69	5,464
1077	28	M	38.1	5.4	SC-ICRC	Cerebrovascular / Stroke	N/A	12	80	97.0	72.4	2.0	9/28/17	256,200	240,125	1.1	7	C = 4 to 6 points	N/A	3,081
1079	61	F	26.9	5.7	SC-ICRC	Anoxia	N/A	9	78	88.0	22.0	1.8	10/9/17	219,117	217,000	1.0	8	B = 7 to 8 points	98.66	4,009
1081	46	M	34.4	5.6	SC-ICRC	Head Trauma	N/A	9	85	97.6	54.8	2.6	10/22/17	600,816	317,000	1.9	9	A = 9 to 10 points	99.58	3,775
1091	34	M	29.3	5.1	SC-ICRC	Head Trauma, Blunt injury	N/A	7	70	96.0	22.8	2.4	11/27/17	151,283	98,500	1.5	7	C = 4 to 6 points	N/A	15,678
1093	59	M	27.2	5.1	SC-ICRC	Cerebrovascular / Stroke	N/A	9	90	97.0	64.9	2.4	12/1/17	264,817	215,500	1.2	8	B = 7 to 8 points	67.38	7,234
1095	64	M	34.6	5.8	SC-ICRC	Head Trauma	N/A	7	80	95.0	88.8	1.5	12/15/17	503,165	354,500	1.4	8	B = 7 to 8 points	99.17	14,628
1096	53	F	27.7	5.5	SC-ICRC	Cerebrovascular / Stroke	N/A	10	80	97.0	32.0	2.6	12/18/17	251,600	251,500	1.0	7	C = 4 to 6 points	95.35	5,945
1097	25	M	24.3	5.1	SC-ICRC	Head Trauma / GSW	N/A	6	70	97.0	52.5	2.4	12/30/17	170,950	114,000	1.5	7	C = 4 to 6 points	82.86	4,306
1098	34	M	33.1	4.8	SC-ICRC	Head Trauma	N/A	4	80	97.0	76.8	2.2	1/4/18	286,950	226,000	1.3	7	C = 4 to 6 points	97.55	3,443
1103	49	M	34.0	6.1	SC-ICRC	Cerebrovascular / Stroke	N/A	8	80	97.0	22.7	2.3	2/13/18	458,600	493,500	0.9	8	B = 7 to 8 points	95.12	16,512
1106	56	M	33.1	5.6	SC-ICRC	Head Trauma	N/A	9	85	94.0	90.3	3.5	3/7/18	237,366	270,500	0.9	8	B = 7 to 8 points	94.77	4,418
1107	45	M	26.5	4.6	SC-ICRC	Cerebrovascular / Stroke	N/A	7	80	94.0	76.4	3.2	3/8/18	594,033	379,000	1.6	8	B = 7 to 8 points	97.88	7,244
1108	30	M	23.3	5.0	SC-ICRC	Head Trauma	N/A	7	73	98.0	38.6	0.8	3/13/18	76,750	96,500	0.8	7	C = 4 to 6 points	88.77	6,894
1115	51	M	30.0	5.7	SC-ICRC	Cerebrovascular / Stroke	N/A	9	70	97.0	N/A	4.9	5/12/18	48,900	48,000	1.0	7	C = 4 to 6 points	81.14	15,184
1125	54	M	23.6	5.8	SC-ICRC	Cerebrovascular / Stroke	N/A	12	83	N/A	27.6	1.6	6/26/18	280,750	210,000	1.3	8	B = 7 to 8 points	N/A	6,467
1126	50	F	23.2	5.6	SC-ICRC	Cerebrovascular / Stroke	N/A	11	80	N/A	11.5	2.2	7/1/18	215,833	274,000	0.8	8	B = 7 to 8 points	N/A	5,282
1130	62	M	31.8	5.8	SC-ICRC	CVA / Stroke	N/A	11	85	97.0	17.9	1.8	7/25/18	264,000	264,000	1.0	8	B = 7 to 8 points	N/A	11,802
1131	29	F	24.1	4.3	SC-ICRC	Cerebrovascular / Stroke	N/A	7	80	98.0	64.6	2.8	8/3/18	255,600	141,500	1.8	8	B = 7 to 8 points	N/A	4,933
1138	25	F	33.5	5.1	SC-ICRC	Anoxia	24	6	75	98.3	31.2	0.7	9/8/18	357,966	245,000	1.5	8	B = 7 to 8 points	N/A	4,237

CVA, cerebral vascular accident; GSW, gunshot wound; ICH, Intracerebral hemorrhage; MVA, motor vehicle accident; SC-ICRC, Southern California Islet Cell Resource Center

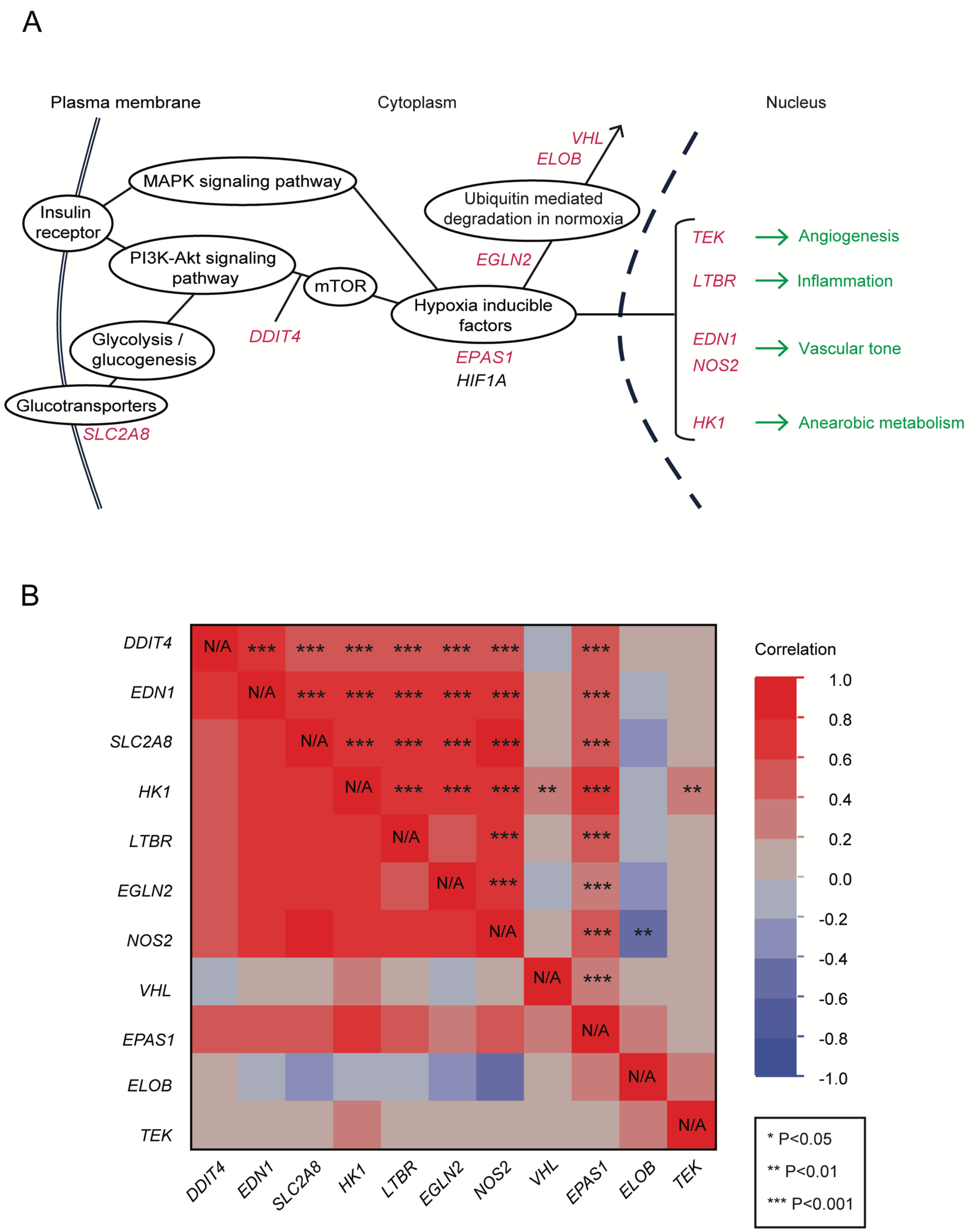

Figure S1. Locations and correlations of the selected genes. (A) The KEGG pathway analysis demonstrates eleven genes mapped to hypoxia-related pathways. Genes demonstrating a significant correlation to the transplantation outcomes are in red. (B) Correlations of 11 genes among our data set of 85 islet batches to each other are presented.

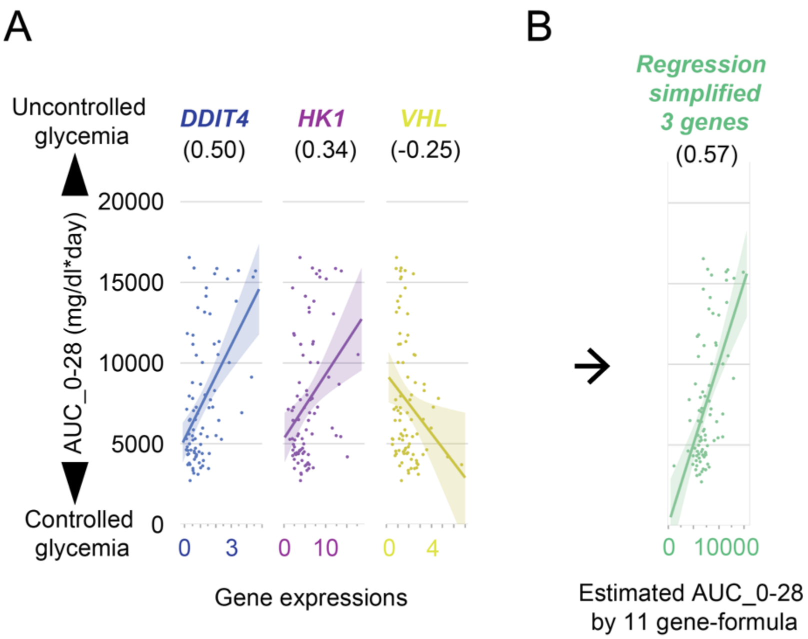

Figure S2. Simplified correlation analyses of the hypoxia-related gene expressions and transplantation outcomes. (A) Correlations of selected 3 key genes to the AUC_0-28. Gene expression is shown in the x-axis, and the transplantation outcome measured using AUC_0-28 is expressed in the y-axis. All islet batches were plotted with the linear regression line. The color bands indicate the confidence of fit at a confidence interval at 95%. Correlations are shown beneath the gene names within parentheses. (B) Simplified multiple regression model using 3 single gene expression data. The Estimated AUC_0_28 value calculated by the linear regression formula (**Table S4**) is expressed in the x-axis.

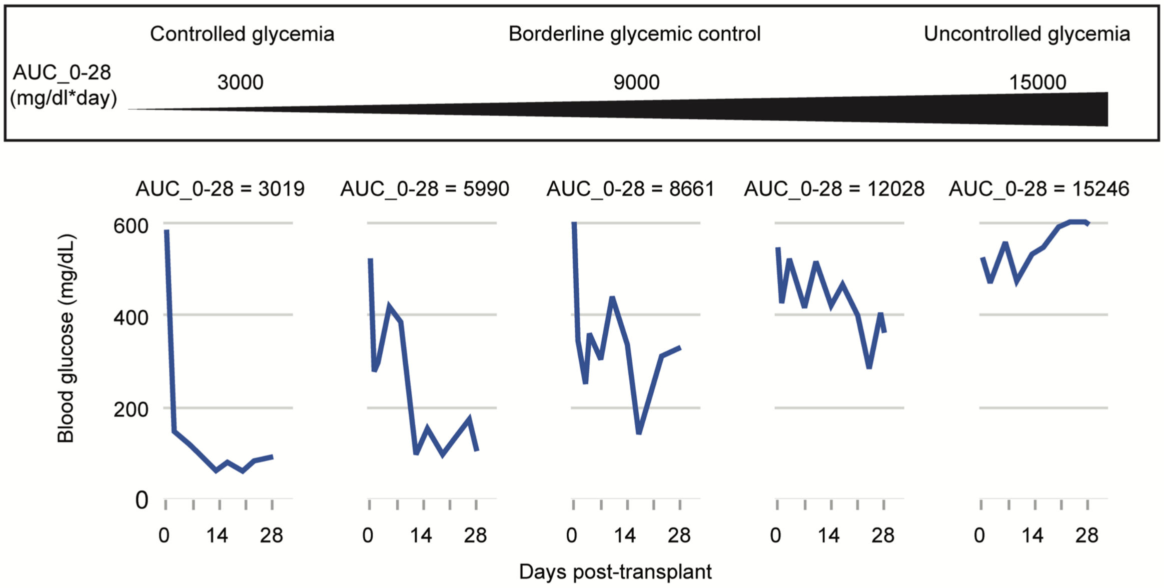

Figure S3. Examples of the area under the curve of the blood glucose for the quantitative assessment (AUC_0-28). Five representative cases of post-transplant mice in our dataset are shown. Cases in the left panels demonstrate well-controlled glycemic control, whereas the right panels demonstrate uncontrolled glycemic control.
